# The impact of prolonged disorders of consciousness on family caregivers’ quality of life – A scoping review

**DOI:** 10.1080/09602011.2021.1922463

**Published:** 2021-06-04

**Authors:** Amy Chinner, Ruth Pauli, Damian Cruse

**Affiliations:** School of Psychology and Centre for Human Brain Health, University of Birmingham, Birmingham, UK

**Keywords:** Disorders of consciousness, Family, Caregiver, Quality of life, Scoping review

## Abstract

Providing long-term care for a family member diagnosed with a Prolonged Disorder of Consciousness (PDoC) can have a significant impact on the lives of family caregivers. This scoping review aimed to explore the current literature investigating the impact of caring for a person in a PDoC on family caregivers’ Quality of Life (QOL), as categorized using the WHOQOL-BREF model. We observed that articles employing quantitative methodologies mostly reported QOL outcomes relating to negative feelings, thinking, learning, memory and concentration, and personal relationships. Articles employing qualitative methodologies mostly reported QOL outcomes relating to negative feelings, personal relationships, positive feelings, and health and social care accessibility and quality. A descriptive content analysis of the QOL outcomes highlighted the limitations of the current literature base in representing the complexities of the experiences of family members providing care for a person in a PDoC. To provide valuable and personalized support to caregivers, without pathologizing or medicalizing their distress, it is vital to characterize more accurately the contextual subtleties of each person’s situation.

## Introduction

Every year in the UK around 10,000 people are admitted to hospital with a moderate or severe head injury (National Institute for Health and Care Excellence, 2014). Due to advances in medical treatments, many of these individuals are now surviving the acute causes of their head injuries. However, a proportion of these survivors subsequently develop a disorder of consciousness (DoC) (Graham et al., 2015) due to their underlying acquired brain injury (ABI). Consciousness is defined as a state comprised of both wakefulness (eyes open and some arousal) and awareness (of the self or the surrounding environment) (Royal College of Physicians, 2020). People displaying an impairment in one or both elements of consciousness will receive a DoC diagnosis, with a diagnosis of prolonged disorder of consciousness (PDoC) currently given in the UK to anyone who remains in a DoC for longer than a month (Royal College of Physicians, 2020). It is estimated that the PDoC incidence and prevalence rates in England and Wales are approximately 5 people per 100,000 a year (Wade, 2018).

The challenges associated with providing long-term care for a loved one with an ABI can have severe adverse implications for family caregivers (Larkin et al., 2018). People recovering from ABIs can require extra support for many years, potentially permanently, with the majority of this support usually provided by unpaid family members (DeJong et al., 1990). Under these circumstances, it is reported that many families make significant personal sacrifices to provide appropriate care for their injured family member (Boschen et al., 2007). This undoubtedly has an impact on the mental health of these family members, with research reporting that a significant proportion of family caregivers of people with traumatic brain injuries (TBI) experience clinically significant emotional distress (Chan et al., 2009).

Although distress responses vary between individuals, it has been found that family caregivers who report a greater burden associated with providing care to a relative post-ABI also report having a significantly poorer health-related quality of life (QOL) (Mar et al., 2011). Similarly, family caregivers of people with more severe behavioural, communication and social difficulties post-brain injury report higher levels of psychological distress (Anderson et al., 2002). Therefore, it is not surprising that a recent systematic review found that many family caregivers of people diagnosed with a PDoC, a state in which individuals have the most severe functional deficits, experience significant changes in their QOL associated with high levels of emotional distress and burden (Soeterik et al., 2017).

While the findings of this quantitative systematic review provided a valuable insight into the psychological distress experienced by caregivers, we know that caring for a person with a brain injury can have complex effects on families’ lives (Larkin et al., 2018). Qualitative research suggests that family members of people diagnosed with a PDoC experience a complicated and conflicting emotional journey, arising from processing the ambiguous clinical situation in which the person lacks consciousness but is still alive (Hamama-Raz et al., 2013). This led the authors of the systematic review to question whether current research is focussing on the “right variables” (Soeterik et al., 2017, p. 1383). Indeed, given the severity of disability that leads to a PDoC diagnosis, it should not be assumed that the impact of being a family caregiver for a person with a PDoC diagnosis is represented by the findings of the general ABI or caregiving literature. To provide the most appropriate and useful support to families of people diagnosed with a PDoC, clinicians must understand the range of psychological, social, and practical outcomes that families experience as caregivers in this context (Kitzinger & Kitzinger, 2014).

To comprehensively address this topic, we conducted a systematic scoping review using an adapted version of the Arksey and O'Malley (2005) scoping study framework as a guide. Scoping reviews enable researchers to map the existing literature in a given area, highlighting central ideas as well as potential gaps (Daudt et al., 2013). This approach allows us to achieve our aim of taking a wide view of the quantitative and qualitative literature, thereby informing future research directions, and supporting an evidence-based understanding of the experiences of family caregivers of people diagnosed with a PDoC. Consequently, our review addresses the following research question: what does the current literature tell us about the impact of PDoC on family caregiver's QOL? Furthermore, what key QOL outcomes have been addressed by researchers in this area, and which outcomes have a more limited, or absent, literature base?

## Materials and methods

We used the Arksey and O'Malley (2005) scoping study framework to guide our review methods, with some suggested modifications as proposed by Levac et al. (2010) and Daudt et al. (2013). This framework consists of six stages: (1) specifying the research question, (2) identifying relevant articles, (3) selecting the applicable articles, (4) extracting the data, (5) collating, summarizing, and reporting the results and (6) optional consultation with key stakeholders (Arksey & O'Malley, 2005). This review was conducted as part of the first author’s Master of Research degree; therefore, a protocol was developed based on the above framework but was not published. We structure this scoping review report in line with the Joanna Briggs Institute format (Peters et al., 2020).

### Eligibility criteria

In keeping with the Arksey and O'Malley (2005) recommendation to maintain a broad review scope, we aimed to comprehensively examine the research literature that investigates the impact of PDoC on family caregiver's QOL. Therefore, article inclusion was not constrained by the type of evidence. There was also no restriction on the publication date for article inclusion. All searches were limited to English language.

To clarify the scope of our review, the key population, concept, and context eligibility criteria were defined as follows.

#### Population

Articles were included in our review if their focal population was adult (aged 18+) family caregivers of people diagnosed with a PDoC. For the purposes of this review, the UK PDoC national clinical guidelines definition of family caregivers was used: “anyone who has a sufficiently close relationship with the patient to be actively concerned with their management and wellbeing” (Royal College of Physicians, 2020, p. 22). This is in line with the inclusion criteria of the related systematic review conducted by Soeterik et al. (2017).

#### Concept

For the purposes of our scoping review, we used the WHOQOL-BREF model (WHOQOL Group, 1998) to guide our definition of QOL. This model proposes multiple facets of QOL in four domains: physical health, psychological, social, and environment. Articles were included in our review if they reported QOL outcomes addressing any of the facets within these four domains. See supplementary materials appendix 1 for the full list of WHOQOL-BREF domains and facets.

#### Context

Articles were included in our review if they focused on family caregiving in the context of a PDoC diagnosis. For the purposes of our review, we used the definition of PDoC provided by the recent Royal College of Physicians (2020) clinical guidelines: a diagnosis given when the person has remained unconscious for more than 4 weeks following a sudden onset ABI. This includes the Vegetative State (VS), Minimally Conscious State (MCS) and Emerging MCS (Royal College of Physicians, 2020). As with Soeterik et al. (2017), articles using traditional or international PDoC diagnostic terminology (e.g., unresponsive wakefulness syndrome) were also included to ensure a comprehensive coverage of the PDoC literature.

As coma is usually considered an acute DoC (Giacino et al., 2014), articles focusing on family caregiving in the context of coma were only included if the PDoC timeframe was met. In the interest of homogeneity, articles investigating family caregivers of people diagnosed with locked-in syndrome, or a DoC caused by a degenerative disease, were not included in our review as these diagnoses are outside of the Royal College of Physicians (2020) definition of PDoC. Healthcare context was not considered during the inclusion or exclusion of articles.

### Search strategy

As recommended by Peters et al. (2020), an initial search was conducted via two online databases; PsycINFO and Web of Science, using broad search terms covering the population, concept, and context of the research question. These terms were chosen through discussion with research experts in this field. The titles, abstracts and index terms of the articles identified in this search were analyzed to determine keywords and phrases to be included as search terms in the full literature search. Based on the number of articles retrieved from the initial searches, we decided to conduct the full search using only the population and context search terms to increase the breadth of coverage.

A full search was subsequently conducted across all the relevant online databases (PsycINFO, Web of Science, PubMed, AMED, CINAHL Plus and Medline) on 30 and 31 July 2020 using the key search terms identified in step 1 (see supplementary materials appendix 2). An example search strategy is provided in supplementary materials appendix 3. An online search was also conducted using the same search terms to identify relevant grey literature, including: theses and dissertations (OATD, OpenThesis, EThOS), conference papers (ZETOC, Web of Science), preprints (medRxiv, preprints.org) and other relevant sources (OpenGrey, Bielefeld Academic Search Engine, Tripdatabase.com). Any additional keywords and phrases identified from relevant articles during these searches were included iteratively as search terms to improve the scope of the review coverage.

The lead reviewer (AC) searched the reference lists of all the articles included in the review for additional unidentified, relevant sources. Due to resource limitations, we were not able to contact the authors of the articles included in the review for further sources of information.

### Source of evidence selection

All search results identified via the above search strategy were exported into Endnote and duplicate entries were removed by the lead reviewer. The remaining articles were reviewed, and selected for inclusion, using our specified eligibility criteria. In line with Peters et al. (2020) scoping review methodology recommendations, two reviewers (AC and RP) completed both stages of article selection independently. Firstly, the titles and abstracts of all the articles were screened, with those not meeting the inclusion criteria excluded at this point. The full text of each article that passed the title/abstract screen was then obtained and examined for eligibility. Articles that passed this full-text screen were included in the review.

**Figure 1.**
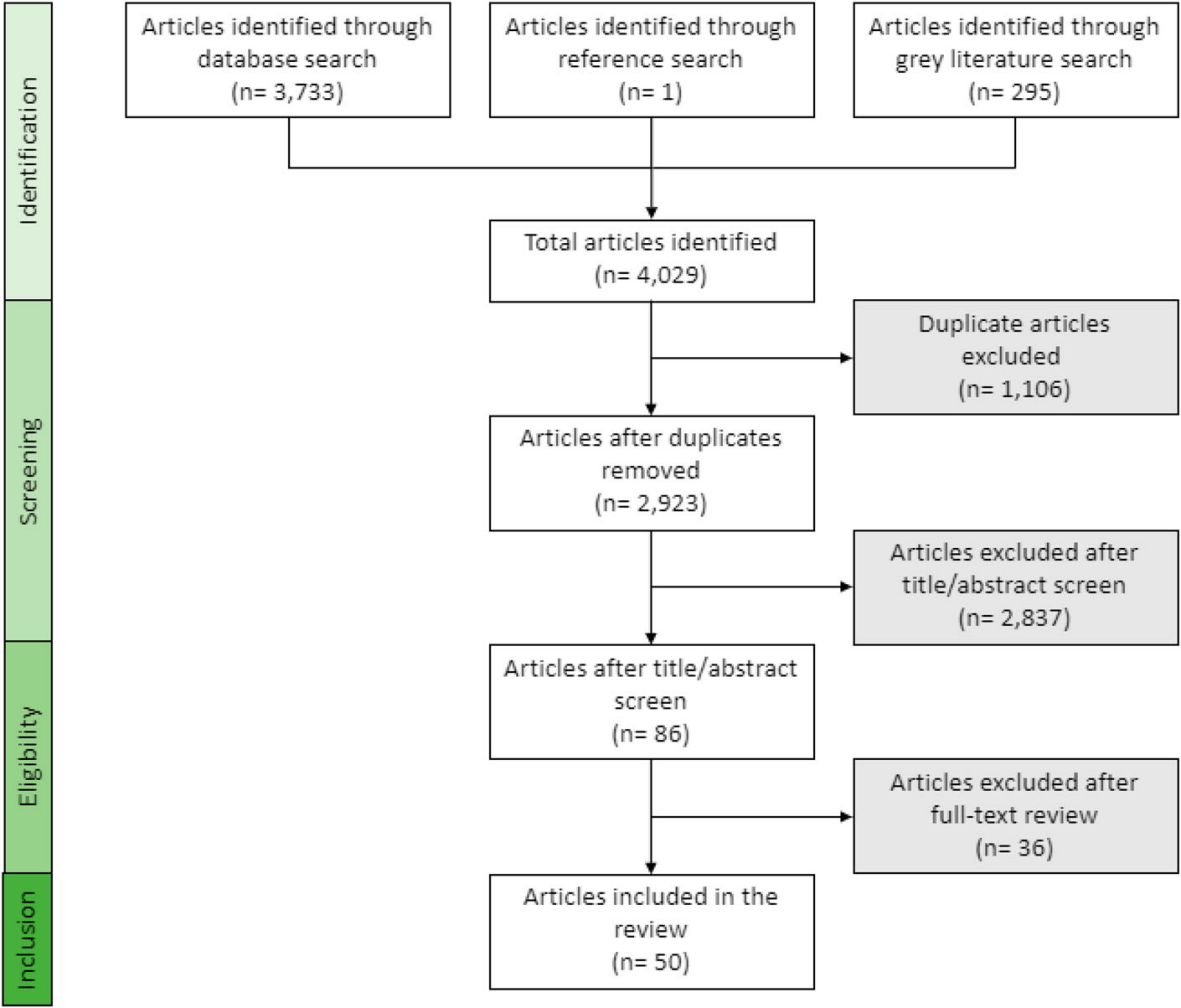
Flow diagram of the source selection process for this scoping review.

Discrepancies after the title/abstract screen were decided based on a comparison of comments provided by both reviewers. Discrepancies after the full-text screen (10.6% of the articles) were discussed by both reviewers to reach a consensus. See Figure 1 for a flow diagram, adapted from the PRISMA statement (Moher et al., 2009), detailing the number of articles included and excluded at each stage of the screening process.

### Data extraction

The necessary data were extracted from the included articles using a data extraction form developed by the lead reviewer (see supplementary materials appendix 4) based on the JBI extraction instrument template (Peters et al., 2020). As recommended by Arksey and O'Malley (2005), the data extraction form was designed to capture general information about the articles (e.g., names of authors, publication year, article type), as well as information directly relating to the research question (e.g., QOL outcomes assessed). The data extraction form was piloted on a small number of articles, and updated to improve functionality, prior to conducting the full search.

It is recommended that at least two reviewers complete the data extraction process (Peters et al., 2020). However, full data extraction by two reviewers was not feasible for this scoping review due to resource limitations. Instead, the lead reviewer extracted data from all the included articles, with the second reviewer (RP) independently extracting data from approximately half (48%) of the articles. Data extracted by each reviewer were compared to ensure replicability. On average the extracted general article data was 89.73% concordant between the reviewers, and the research data was 75% concordant. The lead reviewer then coded the extracted data against the WHOQOL-BREF facets.

### Analysis and presentation of results

We conducted both quantitative and qualitative descriptive analyses of the extracted data as recommended by Arksey and O'Malley (2005). For the quantitative analysis, frequency counts and averages were generated from the extracted article data to provide a detailed summary of the characteristics of the articles included in our review (Levac et al., 2010). To quantitatively report on the concept of our research question (QOL outcomes), frequency counts and percentages were generated to capture the number of articles addressing each outcome domain and facet from the WHOQOL-BREF model (WHOQOL Group, 1998).

For the qualitative summary, a descriptive content analysis was completed based on the method recommended by Sandelowski (2000). Using this approach, we generated key themes reported across the articles using the WHOQOL-BREF domains as a template. This was an iterative process requiring multiple reviews of the extracted data, as well as the original articles. Altogether, this process ensured that the themes generated were driven by the article data itself, adapting the pre-existing WHOQOL-BREF coding system.

As scoping reviews aim to describe, not synthesize, available information (Peters et al., 2020), we deemed the above combination of methodologies to be the most appropriate to provide an overview of the range of research literature available. Unlike systematic reviews, scoping reviews do not aim to provide an assessment of the quality of the articles included (Arksey & O'Malley, 2005). Therefore, we did not conduct any quantitative analyses of article methodological quality for this scoping review.

#### Presentation of the results

We decided to present the results of the above quantitative analyses in tabular and narrative formats for clarity, divided by methodology to aid comparison. Based on Pelentsov et al. (2015), a complete list of all the articles included in our review and their key characteristics is provided in a separate table (see supplementary materials appendix 5 – article reference numbers in superscript below correspond to this table). For the summary of the qualitative content analysis, we divided the narrative into WHOQOL-BREF domains, sub-divided by the generated themes.

## Results

### Characteristics of sources of evidence

#### General article characteristics

In total, 50 articles met the criteria for inclusion in this scoping review. The majority of these were journal articles (*n *= 44) but, due to the wide scope of our review, conference abstracts (*n *= 4), thesis chapters (*n *= 1) and book chapters (*n *= 1) were also included. Half (*n *= 25) of the articles were systematic quantitative studies, the other half of the articles consisted of qualitative studies (*n *= 11), commentaries/opinions (*n *= 8), case studies (*n *= 3), mixed-methods studies (*n *= 2) and a systematic review (*n *= 1). All the articles included in our review were published between 1990 and 2020. Only one article was published in the period 1990–1999, with six published in 2000–2009 and 43 published in 2010–2020. The geographical spread of the articles was not even, with half (*n *= 25) of the articles originating from Italy. This is thought to be due to multiple Italian research groups publishing on this topic, and the existence of two large-scale national Italian studies into VS and MCS (Covelli et al., 2016). The remaining half of the articles originated from nine different countries: UK (*n *= 7), USA (*n *= 4), Germany (*n *= 3), Iran (*n *= 3), Israel (*n *= 2), Norway (*n *= 2), Spain (*n *= 2), Brazil (*n *= 1) and China (*n *= 1).

#### Population characteristics

Most of the articles (*n *= 44) focussed on specific cohorts of family caregivers, ranging from one^12,25,32,38^ to 487^17,27^ caregivers, with some articles reporting on the same cohorts of family caregivers. Five articles did not report on specific cohorts of caregivers; instead, they provided personal reflections on family caregiving in the context of PDoC and/or the existing PDoC caregiving literature base^10,11,24,43,50^. Overall, family caregivers were mostly female and from across the adult lifespan (age range of 18–84). Family caregiver gender was reported in 39 articles, and age in 35 articles; however, caregiver ethnicity was only reported by three articles.

Thirty-eight of the articles reported the family caregivers’ relationship status with the person diagnosed with a PDoC. Of these, approximately 40% of the family caregivers were the individual’s spouse or partner, 20% were the individual’s parent, and 18% were the individual’s child. The remaining ∼22% was made up of siblings, extended family members (e.g., grandparents, nieces, and in-laws), friends and professional caregivers^A^. A small proportion of the articles (*n *= 11) reported the caregiving time provided by the family caregivers – this ranged between 1 hour a week^42^ and 24 hours a day^9,16,19,27^.

#### Context characteristics

Although all the articles considered caregiving in the context of a PDoC diagnosis, only 36 articles reported specific diagnoses. Across these articles, the average percentage of people with a VS diagnosis was 77.1%, and the average percentage of people with an MCS diagnosis was 22.9%. In the 21 articles reporting the cause of the brain injury, the split between TBI and non-traumatic ABI (NT-ABI) varied greatly but overall was roughly equal (average percentage TBI 52.65%, NT-ABI 47.36%). NT-ABI included a range of aetiologies including anoxia, vascular events, and infections. Time post-injury ranged from 1.2 months^9^ to 27 years^9,46^. Healthcare settings included specialist long-term care units, rehabilitation centres and nursing homes, as well as within the family home.

#### Concept characteristics

Across the 50 articles included in our review, QOL outcomes covering all four of the WHOQOL-BREF (WHOQOL Group, 1998) domains were reported. The psychological domain was the most addressed, with almost every article (*n *= 49) reporting at least one psychological outcome. Although the physical health domain was the least frequently addressed, 30 articles still reported on at least one physical health outcome. Negative feelings, personal relationships and financial resources were the facets most reported upon across all the domains.

However, when the articles were divided by their methodologies, the prioritization of facets was not equal. For articles employing quantitative methodologies the facets most reported on were negative feelings, thinking, learning, memory & concentration, and personal relationships. For the articles employing more qualitative methodologies the most reported on facets were negative feelings, personal relationships, positive feelings, and health and social care: accessibility and quality. This suggests a potential disparity between the focus of systematic research in this area, and what caregivers and experts by experience denote as important. See Table 1 for a detailed breakdown of the number of articles addressing each WHOQOL domain and facet.

**Table 1.** Table showing the number of articles addressing each domain, and the percentage of all articles, per article methodology, with outcomes addressing each WHOQOL facet (WHOQOL Group, 1998).

WHOQOL domain (number of articles addressing the domain)	WHOQOL facet	% of all articles addressing each facet (number of articles)	
		Quantitative	Qualitative
Physical health (*n *= 30)	Pain & discomfort	10 (5)	8 (4)
	Sleep & rest	8 (4)	6 (3)
	Energy & fatigue	6 (3)	6 (3)
	Mobility	0	0
	Activities of daily living	6 (3)	14 (7)
	Dependence on medicinal substances/medical aids	4 (2)	0
	Work capacity	22 (11)	16 (8)
	General physical health*	20 (10)	2 (1)
Psychological (*n *= 49)	Positive feelings	2 (1)	34 (17)
	Thinking, learning, memory & concentration	34 (17)	14 (7)
	Self-esteem	0	6 (3)
	Body-image & appearance	0	0
	Negative feelings	52 (26)	40 (20)
	Spirituality/religion/personal beliefs	18 (9)	14 (7)
	General mental health*	24 (12)	8 (4)
Social relationships (*n *= 41)	Personal relationships	34 (17)	40 (20)
	Social support	24 (12)	22 (11)
	Sexual activity	2 (1)	2 (1)
Environment (*n *= 40)	Freedom, physical safety & security	14 (7)	22 (11)
	Home environment	4 (2)	2 (1)
	Financial resources	26 (13)	28 (14)
	Health & social care: accessibility & quality	10 (5)	34 (17)
	Opportunities for acquiring new information & skills	16 (8)	10 (5)
	Participation in recreation/leisure activities	10 (5)	16 (8)
	Physical environment (pollution/noise/traffic/climate)	0	0
	Transport	0	2 (1)
	General environmental QOL*	6 (3)	0

*Additional facets added by the lead reviewer.

It should be noted that each individual article may address more than one facet, both within a domain and across domains. Also, some additional facets were added by the reviewer to capture article results that addressed the domain but did not fall into any specific facet.

### Qualitative summary of sources of evidence

The themes generated from the descriptive content analysis are presented individually below within each WHOQOL domain. See Table 2 for a summary of articles contributing to each theme.

**Table 2.** A summary of the themes identified in the content analysis within each WHOQOL domain (WHOQOL Group, 1998), and the percentage of articles within the domain that contributed to each theme by methodology.

WHOQOL domain (number of articles addressing the domain)	Main themes	% of articles within domain addressing each theme (number of articles)	
		Quantitative	Qualitative
Physical health (*n *= 30)	Caring as a barrier to employment and occupational productivity	40.0 (12)	23.33 (7)
	Physical impact	43.33 (13)	16.67 (5)
	Disrupted daily life	10.0 (3)	30.0 (9)
Psychological (*n *= 49)	Psychological distress and burden	53.06 (26)	20.41 (10)
	Internal resources for coping	28.57 (14)	20.41 (10)
	Loss without death	4.08 (2)	24.49 (12)
	Time as a source of pain and healing	4.08 (2)	16.33 (8)
Social (*n *= 41)	Social support: old and new relationships	46.34 (19)	48.78 (20)
	Changing relationship with their family member	2.44 (1)	21.95 (9)
	Fractious relationships with medical staff	0	14.63 (6)
Environmental (*n *= 40)	A burdensome care system	20.0 (8)	47.5 (19)
	(Lack of) Time for self	25.0 (10)	37.50 (15)
	Financial pressures	25.0 (10)	35.0 (14)

#### Physical health

##### “Caring as a barrier to employment and occupational productivity”

This theme was contributed to by 42.9% of the quantitative articles, compared to 29.2% of the qualitative articles. Due to the broad age spectrum of people with a PDoC, many family caregivers in the articles were within the working age range. However, up to 65.7% of the caregivers reported having to resign from their jobs^19^, either temporarily or permanently, to provide care^9,16,23,24,27,31,33,36,38^. In one longitudinal study caregiver employment status did improve over time, however still only 42.6% of caregivers were in paid employment by the end of the study^9^. Even when caregivers did remain in paid employment, some reported experiencing severe difficulties functioning at work due to the ongoing emotional strain^18,42,44^. As well as affecting the employment of the primary caregiver, two articles reported that caring for an individual in a PDoC impacted significantly on the productivity of the wider family, with siblings dropping out of school to help cover care costs^21,36^.

##### ^“^Physical impact”

Across the 18 quantitative and qualitative articles that contributed to this theme, eight investigated the physical manifestation of caregiver burden via increased psychosomatic^28,50^ and other physical symptoms. These included regular occurrences of insomnia, sleep disturbances and fatigue^5,7,15,21,32^, digestive problems^5^, headaches^5^, eczema^15^ and general physical stress^15,24^. However, caregivers self-reported ratings of their own physical health were variable. In three articles caregivers’ perceived physical health scores, measured using the standardized SF-12 questionnaire (Ware et al., 1996), were in line with population norms^17,19,40^, whereas in another article using the same measure they were reported as significantly lower^27^. In one longitudinal study, the impact of caring on caregivers self-rated health was found to become more of an issue over time^15^. Caregivers’ self-reported health satisfaction ratings were observed in another study using a standardized life satisfaction questionnaire (the FLZ) (Fahrenberg et al., 2000) to vary depending on the healthcare setting, with those caring for people within institutional care settings scoring lower than homecare settings, and lower than the population norm^47^. Two qualitative articles also highlighted the physicality of caring for individuals with such extensive and complex needs as a significant issue for caregivers^32^, with one article reporting physical injuries e.g., backache and arm/knee injuries, caused through the caring role^21^.

##### “Disrupted daily life”

In both the quantitative and qualitative articles, a significant negative impact of caring on caregivers’ daily activities and lifestyle is reported^4,8,15,18,38,42,50^. Due to the prioritization of patient-related activities, caregivers in three articles reported a reduction in their own lives to a point where their basic needs of self-care^15,21^ and nutrition^21,32,50^ were not always met. Adapting to these life changes can be extremely challenging, with one family highlighting the “life abdication” that was required to accommodate their new caring roles (Oliveira et al., 2020, p. 104). However, one longitudinal study suggested that, between 6 and 12-months post-injury, family caregivers begin to re-establish more sustainable daily routines and activities, moving away from a need to live day-to-day^15^. Overall, these issues of disruption and burden on caregivers’ daily lives were raised in 37.5% of the qualitative articles, compared to only 10.7% of the quantitative articles.

#### Psychological

##### “Psychological distress and burden”

Psychological distress was the most investigated outcome across all the articles, but more often this was by articles employing quantitative methodologies. Caregivers scored significantly high on a variety of standardized measures of depressive symptoms in 21 articles, and anxiety in 20 articles, with depression strongly predicting low QOL^45^. Twelve articles also reported increased incidence of Prolonged Grief Disorder (PGD) in cohorts of family caregivers of people with a PDoC using the standardized PGD measure, the PG-12 (Prigerson et al., 2008), with up to 60.4% of caregivers meeting the criteria for this disorder in one study^14^. In the only randomized control trial included in this review, caregivers’ psychological distress, measured using the Symptom Checklist-90-R (SCL-90-R) (Derogatis & Savitz, 1999), was significantly higher than normal at baseline but improved to near normative levels following a single-session group therapeutic intervention^28^. Distress is not limited to the primary caregiver, with significant effects on the mental health of the extended family also reported^21^.

Other psychological symptoms and disorders were also reported by caregivers, including: Post-Traumatic Stress Disorder (PTSD)^22^, anger^20,31,45^, psychophysiological symptoms^28,33,34,35^, hopelessness^41^, guilt^32^, and extreme behavioural responses^15^. One study also investigated the impact of caring for a person in a PDoC on family caregivers’ cognitive function, reporting a negative effect on tasks requiring attention, executive function, and verbal fluency and visuo-spatial memory^35^. Caregivers’ self-reported ratings of their own mental health were lower than the reference norms in three articles^16,27,42^, but two longitudinal studies suggest that caregivers’ self-reported mental health scores improve over time^6,9^. Another study found that caregivers mental health was inversely related to caregiving duration and associated with their family member’s location of residence^17^. The impact of time on emotional burden varied between the articles, with some suggesting that caregivers’ emotional burden worsens over time^1,2^, some suggesting emotional burden lessens over time^9^ and some suggesting it stays roughly the same^45^. One qualitative study reported that the emotional wellbeing of the caregiver echoed the medical state of the person in a PDoC^15^ which may suggest why these results are not consistent.

Using the short form of the Family Strain Questionnaire (FSQ-SF) (Vidotto et al., 2010), the majority of caregivers in two separate studies were assessed to be in need of psychological support, with 46.15%^1^ and 18%^3^ of the respective cohorts reported to be in urgent need. When asked about access to psychological support, 34.3% of caregivers in one study reported having access^9^, and 17.6% of caregivers in another study reported having asked for psychological support within the first year of caregiving^4^. Although many caregivers may, and indeed do, benefit from professional psychological help, it must be noted that the pathologization and medicalization of legitimate responses to a traumatic situation was also reported to increase caregivers’ distress^25,26^.

##### “Internal resources for coping”

The quantitative studies tended to focus on investigating the coping strategies employed by family caregivers using various versions of the COPE questionnaire (Carver, 1997; Sica et al., 1997). Overall, caregivers used problem-focussed and emotion-focussed coping strategies most frequently^3,9,14,16,19,27,33,45^, with avoidance/disengagement and humour used least frequently^3,9,13,27,33^. One study suggested that this may vary based on the diagnosis, with caregivers of people in a VS using avoidant coping strategies significantly more than caregivers of people in a MCS^17^, but another found no differences between caregiver groups^47^. Significant correlations were also reported between psychological distress and types of coping strategy used. Greater burden was positively associated with avoidance^9^, and anxiety, depression and PGD were positively associated with denial and self-blame^13,14,45^, but negatively associated with acceptance^13,14^. However, one study did not find any correlation between coping strategies and emotional burden^42^.

Only qualitative articles highlighted caregivers’ discovery of an unknown internal strength that enabled them to cope with the process of caring and the positive impact that this had on their self-esteem^8,10,18,23,25,30^. Religion as a coping strategy was referred to in both quantitative and qualitative articles. Three articles reported that faith helped caregivers cope and reason with their current situation^32,38,50^, with another reporting that use of spiritual support was associated with fewer sleep disturbances^7^. However, in two articles caregivers reported that religion either provided them no support^2^ or they did not use it as a strategy at all^36^.

##### “Loss without death”

A strong theme emerging from the qualitative articles was the complicated emotional processing that caregivers endure as a result of experiencing a bereavement without the ability to mourn^10,23,24^. Caregivers’ report struggling with conflicting emotions associated with a “living loss” (Crow, 2006, p. 185), including sorrow at the loss of the person the individual was before, while still hoping for improvements and feeling guilty when this hope diminishes^20,23,25^. Caregivers in one article described the situation as “worse than a death” (Soeterik et al., 2018, p. 1398) due to the ambiguous prognosis and constant threat of medical complications and death. In two quantitative studies, caregivers were reported to experience significant difficulties with making sense of the meaning of their own life in the context of the person’s diagnosis and ongoing condition^44,47^. These findings are consistent with the theory of ambiguous loss (Boss, 2007) where uncertainty about an individual’s state of presence obstructs grief and decision-making processes for those close to them.

The situation is further complicated by the difference in perceptions and reactions between family caregivers and medical professionals^30,36^, or between family members^10^, which can cause significant frustration, anger, and feelings of abandonment. These conflicts often arise around decisions regarding the continuation or withdrawal of life-sustaining treatments. One article reports significant distress caused to caregivers by the artificial medical maintenance of the person in a state they would not wish to be in^26^. However, in another article, five caregivers reported wanting to continue with life-sustaining treatments, regardless of what they thought the individual would want, rather than face losing them^4^.

##### “Time as a source of pain and healing”

Qualitatively, time was first seen as a source of pain for caregivers, with many articles reporting that caregivers lived day-to-day and avoided thinking about the future^8,18,23,46^. This enabled them to cope with the ongoing emotional challenges of the diagnosis and caregiving, supported by hope for a positive outcome. Generally, increasing time post-diagnosis led to an abandonment of hope for recovery^10,20^. One article reported that caregivers’ initial hope for a good outcome decreased at around 4–6 months post-injury^11^, and another reported that 45.8% of caregivers lost hope in the first year of caregiving^4^. For some caregivers, this decline in hope led to feelings of guilt at having given up on the individual^12,25^, but others reported shifting to smaller hopes for more attainable outcomes^29,30^.

As caregivers’ perspectives on the person’s prognosis changed, their realization of the situation resulted in feelings of devastation^25^, but also acceptance^29,32^. This acceptance allowed caregivers to start a process of renormalization in their daily lives, but also led to feelings of guilt at spending less time with their family member^15^. Quantitatively, caregivers’ overall needs, measured using the Caregivers Needs Assessment (Moroni et al., 2008), were reported to decrease over time, but only for caregivers of people in a VS^6^. Caregivers’ overall QOL was also reported to decrease over time^1^.

#### Social

##### “Social support: old and new relationships”

Caregivers’ need for, and sources of, social support were covered both quantitatively and qualitatively by almost every article in this domain. The deterioration of personal relationships was highlighted as a key issue^4,8,18^, and relationship quality was rated as significantly lower than normal in three articles using standardized measures^2,16,17^. Interpersonal hostility and sensitivity were reported to be high in seven articles both quantitatively using the SCL-90-R (Derogatis & Savitz, 1999)^7,28^, and qualitatively^21,50^. This was a particular issue within families due to differing emotional reactions to the person’s diagnosis^10,20^ and the prioritization of patient-related activities^30^. Some caregivers also reported the noticeable impact of their own coping on their wider family^4^, sometimes leading to the primary caregiver hiding their emotions from their family to protect them^23^.

Problems with social involvement and functioning were also described as a significant issue for caregivers^2,5,9,27,33,42,44^, with over 70% of caregivers in one article reporting a lack of opportunities to socialize^2^. Over time this led to increased social isolation for caregivers^10,21,36^ and loss of friendships^4,8,24^. The decline in social involvement was positively correlated with caregivers’ depressive symptoms^2,9^, and with their family members living in institutional care settings^4,17^. Caregivers in three articles also highlighted the negative impact of caregiving on their social status and identity, with the loss of their previous roles at work and at home^18,24,26^.

The support of relatives was highlighted as crucial in allowing caregivers to begin to renormalize their lives^15,30^, with relatives providing a source of strength^23,37^ and sharing the caring burden^12,32^. Caregivers also developed new supportive relationships with other families experiencing similar situations, or more formally with care staff^8,10,24^. However, when assessed quantitively using the COPE (Sica et al., 1997), even though social support was found to be a useful coping strategy, it was not the most used by caregivers^16,17^. This may be in part due to a reported decline in social support over time^37,38,45,50^, especially following discharge from hospital^48^, therefore making it an unreliable resource. Perceived social support scores did not vary between caregivers of people with a PDoC or other chronic conditions in one study^16^, suggesting that this may be an issue for caregivers more generally.

##### “Changing relationship with their family member”

Caregivers qualitatively described the initial need to be physically close to the person with a PDoC to be able to sustain their emotional connection, and to communicate their needs^4,18,20,24^. This allows caregivers to maintain their bonds with their family member^8,44^, upholding the importance of their relationship through continued commitment and love^23^, which some saw as their duty^4,32^. For some caregivers, part of this duty was to ensure that the individual continued to receive social visitors^30^, or that their absence in other relationships was compensated for by the caregiver^18,46^ in order to uphold the person’s continued existence.

Over time, the nature of caregivers’ relationship with their family member was reported to adapt to their new roles, with many non-parent caregivers reporting to now see the person in a PDoC as a child-like figure^8,18,23^. Some caregivers expressed a nostalgia for their past relationship^8^, often due to the loss of their main emotional support figure^12,23^ and the un-reciprocal nature of the new relationship^46^. Caregivers’ need for physical closeness with the individual in a PDoC was reported to lessen over time^15^. In the only quantitative study addressing this topic it was found, using the adapted Boundary Ambiguity Scale (Soeterik, 2017), that all caregivers had high confusion about their new relationship with their family member, and 54.5% of caregivers were unsure of the role the person now had within the family unit^44^.

##### “Fractious relationships with medical staff”

This theme was only reported on by qualitative articles. Clashing opinions about the medical condition and prognosis of the person in a PDoC were reported to lead to the development of stressful, negative relationships between family caregivers and clinical teams^10,11,30^. For some caregivers, deterioration of this relationship causes a distrust and avoidance of medical staff^4,18,29^. However, other caregivers report experiencing positive relationships with staff, from which they receive significant emotional and practical support^8,37^. One article suggests that caregivers’ relationships with staff reflect their adjustment to their family member’s prognosis^10^. However, as this puts the responsibility of relationship solely on the caregiver, we suggest that this is too simple a view of these complex relationships.

#### Environmental

##### “A burdensome care system”

The largest proportion of articles contributing to this theme were qualitative, with 79.2% of the qualitative articles reporting at least one outcome relating to systemically caused burden. In these articles, many caregivers expressed a need for simplified care pathways, with the requirement to navigate complicated, bureaucratic systems to advocate for their family member’s needs being reported as a cause of excessive burden and strain^8,24,48^. The system made it challenging for caregivers to attain, and trust that the person in a PDoC would receive, suitable continuity of care^26,48^. This was heightened by a reported lack of access to medical teams^12,36,37^, adequate care resources^32,37,48^ and respite/support services^16,48^. However, some caregivers did report having access to good professional and peer support services^5,9,23,38^.

Challenges communicating with health and social care services^29,46^ and negative experiences of uncaring communications from medical staff^4^ also added to caregivers’ difficulties. Good communication between staff and caregivers through the longitudinal process of advance care planning was reported as vital in supporting families to understand, and cope with, a PDoC diagnosis^43^. Caregivers in one article highlighted their view of staff nurses not only as vital care facilitators but also educators^37^. However, in the quantitative studies, caregivers’ scores on the FSQ (Rossi Ferrario et al., 1998) indicated an elevated need for knowledge and information^5,9,17,27,33,35^, suggesting that ongoing communication with staff may not be happening for all caregivers. One study did find that caregivers informational need decreased over time^9^, but another found no change in this need over 8 months^33^.

##### “(lack of) time for self”

In both quantitative and qualitative articles, the negative impact of caregiving on caregivers’ personal interests was reported^8,15,20,27,38,42^. In one study, 62% of caregivers reported a lack of time for themselves^5^, which echoed the findings from two qualitative articles^8,18^. Some caregivers reported a lack of opportunity to engage in outdoor and leisure activities^2^ and low engagement with previous hobbies^10^. However, in one longitudinal study, the negative impact on caregiver’s self-reported leisure activities was found to be less severe at 12 months post-injury than at six months^15^. Caregivers in another study reported no change in their physical activity over time^6^.

Qualitatively, caregivers highlighted their experience of “domestic imprisonment” (Goudarzi et al., 2015, p. 4) due to caring becoming their only purpose in life^12,26^. This issue of time spent providing care was also investigated quantitatively where it was found that caregivers reported spending large numbers of hours providing care every day^16,36^, and this significantly predicted overall caregiver burden^17,45^. Lack of freedom was particularly prominent for home-caregivers^47^, and approximately 25% of caregivers in two studies reported that they had taken on the caring role because no-one else was able to do so^27,33^. Despite this huge amount of personal sacrifice, caregivers in two qualitative studies reported that for them caring was not seen as an obligation but something they wanted to do for their family member^23,38^.

##### “Financial pressures”

A strong theme across quantitative and qualitative articles was the negative impact of caring on caregivers’ financial situations^5,8,9,16,19,21,27,29,36,45^. In one study 50% of caregivers reported having experienced economic problems^2^, with two other articles reporting that caregivers experienced reduced incomes due to their caring roles^23,24^. In two Italian studies, between 40% and 47% of families reported incomes below the national average of €17,000 a year^27,42^. However, when asked to rate their own economic status on a Likert scale, over 80% of caregivers in six Italian and Spanish studies rated their financial situation as average or above average^9,13,14,19,27,33^. This was supported by another German study, which reported that caregivers’ self-reported satisfaction with their financial situation on the FLZ (Fahrenberg et al., 2000) was within the normal range^47^. On the other hand, it has also been reported that caregivers’ self-rated financial concerns increase over time^1^, so one-off ratings may not be representative long-term. The financial burden of caring for a person with a PDoC was also shown to extend beyond the primary caregiver, with other family members stepping in to provide financial support^21,37,38^.

Reasons for caregivers’ financial difficulties were reported qualitatively. This included the loss of the family breadwinner and the caregiver becoming the sole earner^10,38^. Another issue was the high costs of caring for people with extensive medical needs^20,21,38^. This was particularly challenging for those providing care at home^32^, and for caregivers reliant on medical insurance^32,36^ as the insurance cover was not always sufficient, leading to medical debt. Despite these difficulties, only around 15% of the caregivers in two quantitative studies reported receiving economic aid^16,33^. Qualitatively, one caregiver discussed the necessary prioritization of the family finances, often having to make personal sacrifices, to ensure their family member received the right care^38^. However, in another article where financial problems were regularly mentioned by caregivers, negatively rated QOL was not perceived as related to wealth^11^.

## Discussion

Through this scoping review, we have identified a wide range of psychological, social, and practical QOL outcomes that families experience as caregivers of people with a PDoC. This supports the argument that to determine the best support for each caregiver in this situation, we need to understand the context in which care is provided (Kitzinger & Kitzinger, 2014). However, despite the quantity and variety of articles included in this review, it is likely that the systematic literature addressing caregiver QOL in the context of PDoC is limited to preconceived areas of importance and therefore not representative of the true clinical picture.

It is frequently documented in the quantitative literature that having a family member diagnosed with a PDoC is an extremely traumatic event, with many caregivers shown to experience high levels of psychological distress (Soeterik et al., 2017). Although the negative impact of caring on psychological QOL is a strong theme in the qualitative literature too, caregivers also discuss some positive psychological aspects. This included recognizing their own inner strength and ability to cope, which can lead to improvements in self-esteem, as well as seeing caring as a way to continue to show love and commitment to their family member. These positive outcomes should not be overlooked when systematically researching QOL impact, as understanding the factors that may be protective or motivational is paramount to a person-centred psychological formulation (Division of Clinical Psychology, 2011), from which appropriate support structures can be developed.

Similarly, although the quantitative literature did address some of the everyday difficulties experienced by caregivers, the themes focussing on practical issues (e.g., disrupted daily living, and themes in the environmental domain) were more frequently addressed qualitatively. This was particularly noticeable regarding the distress caused to caregivers by the healthcare system itself. Qualitatively, many caregivers reported significant burden associated with navigating complex, bureaucratic systems, and fighting to access necessary care and support. However, quantitatively, this was only investigated via caregivers self-reported informational needs. This divergence in focus between the article methodologies indicates that researchers, and perhaps clinicians, perceive caregivers in a much more passive role within the care system than the caregivers themselves.

It is important that future systematic research addresses caregivers’ needs for practical support as active participants within the care system. Developing capable environments (McGill et al., 2014) that support caregivers throughout their caring journey, rather than just focussing on points of psychological crises, will enable a more proactive approach to improving caregivers’ QOL and help to reduce the pathologization of their legitimate distress responses (Kitzinger & Kitzinger, 2014). It is worth noting that the majority of the quantitative articles included in our review were Italian (*n *= 20), in line with the previous review by Soeterik et al. (2017), whereas the qualitative articles were more geographically mixed. Due to the varying healthcare contexts and cultural expectations of caregiving across countries, further research needs to be undertaken to understand the specific QOL impacts and caregiver needs within local contexts of health and community care services.

It is also important to mention that most of the quantitative articles included in this review employed self-report measures. Although this allows a systematic approach to investigating caregivers’ perceptions of their own situation, the results are potentially impacted by limited response options and subject bias. The combination of these errors may mask the true impact of caring in these studies. For example, quantitatively, caregivers’ self-rated financial status indicated that they perceived their financial situation as average or above (Covelli et al., 2016). However, qualitatively, caregivers reported experiencing significant financial difficulties due to caring for a person with extensive medical needs (Oliveira et al., 2020). Similarly, caregivers were often asked to quantitatively rate their perceived physical health, but the physicality of providing care was only reported qualitatively (Goudarzi et al., 2015; Martone, 2000). Therefore, it is important to consider how the question asked may lead to significantly different results, which in turn could impact on the perceived support needs of a specific group.

Similarly, it is important to consider who is represented by the research being conducted. Contextual factors such as ethnicity and socioeconomic status were reported on infrequently (or not at all) by the articles include in our review. However, these factors provide significant context to the experiences of caregivers. For example, Black Asian and Minority Ethnic (BAME) caregivers have been reported to face significantly more health challenges than their white counterparts (Carers UK, 2011). By insufficiently exploring these contextual aspects, the lessons that we take from the literature will be limited and could result in serious underestimations or misrepresentations of caregivers’ needs.

Finally, it may be beneficial for future research in this area to focus more on caregivers’ needs rather than just QOL outcomes. By applying a needs-based template, such as the Supportive Care Needs Framework (Pelentsov et al., 2015), researchers could help develop practical, person-centred guidance on how to best support families providing long-term care for relatives in a PDoC.

### Limitations of this review

Altogether we found that the WHOQOL-BREF (WHOQOL Group, 1998) was an acceptable model to capture the breadth of QOL outcomes experienced by caregivers of people in a PDoC, and a useful template for guiding this review. However, using a template in this way does potentially limit the scope of the analysis beyond the pre-existing model. For example, we felt that some of our themes addressed key issues that crossed multiple domains e.g., fractious relationships with medical staff could be usefully included within a burdensome care system. These inter-domain relationships were similarly highlighted by the significant number of correlations between QOL outcomes in different domains e.g., the association between depressive symptoms and perceived economic problems (Magnani et al., 2020). This suggests that the lack of representation of interactional pathways between domains in the WHOQOL-BREF model may be too reductionist.

Future research, and clinical practice, would benefit from exploring the interaction between factors affecting caregivers’ QOL. As most of the articles in our review employed cross-sectional approaches, a key part of this will be improving the investigation of the longitudinal impact of caring on family caregivers of people in a PDoC. This will be particularly important given the large variation in time post-injury in the articles included in our review, which could impact on the relative importance of different risk or protective factors. Additionally, due to the international nature of the articles included in our review, our results are not specifically reflective of local, religious, health and social care or funding contexts. Understanding the development of QOL outcomes over time within the specific caregiving context is paramount given the prolonged nature of the disorder, and the likely significant impact of the local context on PDoC service availability and expectations of the role of the caregiver.

Due to practical limitations for this scoping study, we were unable to formally conduct the optional sixth stage of the Arksey and O'Malley (2005) framework: consultation with relevant stakeholders. Therefore, our interpretations may be limited by our own perceptions and preconceptions. We tried to minimize the effect of this limitation by consulting with research colleagues who have expertize working with people in a PDoC to shape an appropriate review focus and scope. However, future research in this area would benefit from consultation with family caregivers, as well as clinical staff working in this area.

Although the Arksey and O'Malley (2005) framework dismisses quality assessment as a necessary part of a scoping review, it has been argued that this limits the ability to comment on the clinical implications of scoping review results (Daudt et al., 2013). As the purpose of our review was to map the research literature in a specific area, and not necessarily to provide clinical recommendations, we did not feel that a quality assessment of included articles was essential. However, it would be recommended that any future research assessing literature quality in this area employs both quantitative and qualitative quality assessment tools due to the highly diverse article methodologies addressing this topic.

As with any literature review, scoping reviews are limited by the availability of relevant sources of information (Peters et al., 2020). Although we did include many diverse articles in our review, it is possible that by focusing our research question on understanding the academic literature we could have missed important sources of alternative information (e.g., narrative accounts published in popular literature). Similarly, due to our stringent inclusion and exclusion criteria, we excluded some articles that could have provided useful additional information (e.g., caregiving in the context of PDoC caused by a degenerative disease, or articles not reported in English). Although this provided the homogeneity of articles needed to address our research question, this may not accurately reflect the entire clinical picture.

## Conclusion

The current literature demonstrates that caring for a person diagnosed with a PDoC can have a wide-ranging and considerable impact on family caregiver's QOL. However, to date, most research in this area has consisted of systematic quantitative investigations of the negative consequences of caring on caregivers’ physical and mental health. This limited focus and methodology overlooks significant complexities in caregivers’ emotional and practical experiences. Understanding these sometimes subtle, contextually sensitive occurrences is vital in providing personalized support to caregivers without pathologizing or medicalizing their distress.

Altogether, our scoping review highlights the need for future research to take a more systemic view of caregiving within the context of a PDoC diagnosis. Changing the focus of future research in this way will allow us to improve three key areas of understanding: the relational interactions between different QOL outcomes (including how these relationships change over time), the contextual and cultural factors influencing QOL outcomes, and the practical needs of caregivers within the caregiving system.

## Supplementary Material

Supplemental Material

## Note

1 A. Two articles including professional caregivers in their cohorts were accepted for inclusion in this review as the focus was deemed to be sufficiently on the impact of PDoC on family caregivers to meet the inclusion criteria.
