# Supplementary material for: The impact of prolonged disorders of consciousness on family caregivers’ quality of life – A scoping review
Source: Neuropsychol Rehabil. 2021 Jun 4;32(7):1643–66. doi: 10.1080/09602011.2021.1922463 (PMC9487862; doi:10.1080/09602011.2021.1922463)
Supplement: Supplemental Material [file PNRH_A_1922463_SM2373.pdf]

Supplementary Material - Appendices

Appendix 1: List of domains and facets taken from the WHOQOL-BREF model of Quality of Life (WHOQOL Group, 1998), and the facet coding system used for this scoping review.

WHOQOL-BREF Domain	WHOQOL-BREF Facet	Facet code
Physical Health	Pain & discomfort	101
	Sleep & rest	102
	Energy & fatigue	103
	Mobility	104
	Activities of Daily Living (ADLs)	105
	Dependence on medicinal substances/ medical aids	106
	Work capacity	107
Psychological	Positive feelings	201
	Thinking, learning, memory & concentration	202
	Self-esteem	203
	Body-image & appearance	204
	Negative feelings	205
	Spirituality/religion/personal beliefs	206
Social	Personal relationships	301
	Social support	302
	Sexual activity	303
Environmental	Freedom, physical safety & security	401
	Home environment	402
	Financial resources	403
	Health & social care: accessibility & quality	404
	Opportunities for acquiring new information & skills	405
	Participation in recreation/leisure activities	406
	Physical environment (pollution/noise/traffic/climate)	407
	Transport	408

Appendix 2: List of search terms used for the main search of the research and grey literature databases divided by research population and context.

**Population: Family caregivers**	**Context: PDoC**
Famil*<br>(includes includes family, families, family members, familial)	Disorder* ADJ2 conscious*<br>(includes disorder/s of consciousness, prolonged DOC, post-traumatic DOC, disordered consciousness)
Caregiv*<br>(includes caregivers, caregiver, and caregiving)	Unresponsive wakeful*<br>(includes unresponsive wakefulness syndrome)
Next of kin	Minimal* ADJ conscious*<br>(includes minimally conscious, minimal consciousness and minimally conscious state)
	Vegetative state
	Apallic
	Unawareness state
	Low awareness state
	Covert ADJ2 conscious*
	Post-coma unawareness

Appendix 3: Example search strategy used for Web of Science search 31/07/2020 (Web of Science Core Collection and Medline), and the number of results received.

#	Search terms	Results
1	TS=((disorder* near/2 conscious*) or (unresponsive near/2 wakeful*) or (minimal* near/2 conscious*) or "vegetative state" or (covert near/2 conscious*) or "post-coma unawareness" or apallic or "unawareness state" or "low awareness state")<br>Databases= WOS, MEDLINE Timespan=All years<br>Search language=Auto	11,686
2	TS=(famil* or caregiv* or “next of kin”)<br>Databases= WOS, MEDLINE Timespan=All years<br>Search language=Auto	2,318,532
3	#2 AND #1<br>Databases= WOS, MEDLINE Timespan=All years<br>Search language=Auto	1,122
4	#2 AND #1<br>Refined by: LANGUAGES: (ENGLISH)<br>Databases= WOS, MEDLINE Timespan=All years<br>Search language=Auto	1,037
5	#2 AND #1<br>Refined by: LANGUAGES: (ENGLISH) AND DOCUMENT TYPES: (ARTICLE OR ABSTRACT OR OTHER OR CLINICAL TRIAL OR CASE REPORT OR REVIEW)<br>Databases= WOS, MEDLINE Timespan=All years<br>Search language=Auto	932

Appendix 4: Example data extraction form detailing the data collected for each article, including the expected data format.

General data to be extracted		Expected data format
General article details	Authors	Text
	Publication year	Number
	Study aim	Text
	Country (determined from lead author address)	Text (determined from lead author correspondence address)
	Article type	Text (e.g., journal article, thesis)
	Study design	Text (e.g., qualitative study, systematic review)
	Study methods	Text (e.g., in-person semi-structed interviews, named standardised questionnaire/s)
	Number of participants	Number
Caregiver-related details	Age	Numerical range
	Gender	Text (reported groups) & number (frequencies)
	Ethnicity	Text (reported groups) & number (frequencies)
	Education Level	Text (reported groups) & number (frequencies)
	Employment status	Text (reported groups) & number (frequencies)
	Marital status	Text (reported groups) & number (frequencies)
	Relationship with the patient	Text (reported groups) & number (frequencies)
	Primary caregiver?	Text (Y/N) & number (frequencies)
	Caring time provided	Text (reported groups) & number (frequencies)
Patient-related details	Age	Numerical range
	Gender	Text (reported groups) & number (frequencies)
	Patient's PDoC diagnosis	Text (reported groups) & number (frequencies)
	Causal brain injury	Text (reported groups) & number (frequencies)
	Time post-injury	Numerical range
	Healthcare context	Text (reported groups) & number (frequencies)
**Research question data to be extracted**		**Expected data format**
Domain: Physical health	Facets addressed	Facet code/s or 0
	Quant results	Text & number, or blank
	Qual results	Text (reported themes) or blank
Domain: Psychological	Facets addressed	Facet code/s or 0
	Quant results	Text & number, or blank

	Qual results	Text (reported themes) or blank
Domain: Social	Facets addressed	Facet code/s or 0
	Quant results	Text & number, or blank
	Qual results	Text (reported themes) or blank
Domain: Environmental	Facets addressed	Facet code/s or 0
	Quant	Text & number, or blank
	Qual results (themes)	Text (reported themes) or blank

<u>Appendix 5</u>: Full list of all the articles included in this scoping review.

**Article number**	**Paper Reference**	**Country**	**Study/Article Aim**	**Article Type**	**Study Design**	**Data collection methods**	**Number of caregivers (M:F)**	**Caregiver age (range/mean years)**	**WHOQOL BREF domains addressed**
1	Bastianelli, A., Gius, E., & Cipolletta, S. (2016). Changes over time in the quality of life, prolonged grief and family strain of family caregivers of patients in vegetative state: A pilot study. *Journal of Health Psychology, 21*(5), 844-852.	Italy	To explore changes over time in the self-reported quality of life, prolonged grief, and family strain of informal caregivers of patients in a VS.	Journal article	Quantitative study - observational (longitudinal)	4 self-reported questionnaires: ADSS, PG-12, FSQ & CQOL (completed at baseline and 12m - ADSS baseline only)	52 (22:30)	19-85	P, S, E
2	Chiambretto, P., Ferrario, S., & Zotti, A. (2001). Patients in a persistent vegetative state: Caregiver attitudes and reactions. *Acta Neurologica Scandinavica, 104*(6), 364-368.	Italy	To investigate the emotional reactions, psychosocial aspects, and coping styles of DOC caregivers in Italy.	Journal article	Mixed methods study - observational (cross-sectional)	5 self-reported questionnaires: STAI X1 X2, EPQ, QD, CISS & FSQ2<br>Semi-structured interviews (part of FSQ2)	16 (6:10)	25-75	P, S, E
3	Cipolletta, S., Gius, E., & Bastianelli, A. (2014). How the burden of caring for a patient in a vegetative state changes in relation to different coping strategies. *Brain Injury, 28*(1), 92-96.	Italy	To understand the experiences of caregivers of patients in a VS and to differentiate their experience based on their different levels of anxiety, depression and family strains.	Journal article	Quantitative study - observational (cross-sectional)	3 self-reported questionnaires: ADSS, PG-12, FSQ-SF & COPE	61 (23:38)	18-85	P, S
4	Cipolletta, S., Pasi, M., & Avesani, R. (2016). Vita tua, mors mea: The experience of family caregivers of patients in a vegetative state. *Journal of Health Psychology, 21*(7), 1197-1206.	Italy	To understand whether, and how, caregivers of patients in a VS experience an emotional paradox between life and death.	Journal article	Qualitative study	Semi-structured interviews – Interpretative Phenomenological Analysis	24 (5:19)	32-70	PH, P, S, E
5	Corallo, F., Bonanno, L., De Salvo, S., Giorgio, A., Rifici, C., Lo Buono, V., Bramanti, P., & Marino, S. (2015). Effects of Counseling on Psychological Measures in Caregivers of Patients with Disorders of	Italy	To evaluate the psychological impact on caregivers after the diagnosis of their family member with a DOC, and	Journal article	Quantitative study - observational (cross-sectional)	2 self-reported questionnaires: FSQ & SCL-90-R	50 (24:26)	Mean 52.88 (SD 11.61)	PH, P, S, E

	Consciousness. *Am J Health Behav, 39*(6), 772-778.		whether these vary according to the type of diagnosis (VS and MCS).						
6	Corallo, F., Bonanno, L., Lo Buono, V., De Salvo, S., Allone, C., Palmeri, R., . . . Marino, S. (2017). Evolution of psychological condition in caregivers of patients with disorders of consciousness: A longitudinal study. *Neurological Sciences, 38*(7), 1249-1253	Italy	To assess the evolution of mood disorders, especially anxiety and depression, in caregivers of patients with DOC during the hospitalization of their relatives, and to evaluate changes in caregivers' needs.	Journal article	Quantitative study - observational (longitudinal)	5 self-reported questionnaires: BDI-II, STAI-Y, SF-36, PG-12 & CNA (completed at baseline and 6m)	80 (26:54)	Mean 45.20 (SD 14.24)	PH, P, S, E
7	Corallo, F., Bonanno, L., Lo Buono, V., De Salvo, S., Rifici, C., Bramanti, A., & Marino, S. (2018). Coping strategies in caregivers of disorders of consciousness patients. *Neurological Sciences, 39*(8), 1375-1381.	Italy	To compare distress and coping strategies used among caregivers of patients in a DOC.	Journal article	Quantitative study - observational (cross-sectional)	2 self-reported questionnaires: SCL-90-R & COPE	80 (30:50)	Mean 47.87 (SD 11.28)	PH, P, S
8	Covelli, V., Cerniauskaite, M., Leonardi, M., Sattin, D., Raggi, A., & Giovannetti, A. M. (2014). A qualitative study on perceptions of changes reported by caregivers of patients in vegetative state and minimally conscious state: the "time gap experience". *ScientificWorldJournal, 2014*, 657321.	Italy	To understand how female informal caregivers of patients in a DOC describe the life changes experienced after the acute event of their family member.	Journal article	Qualitative study	Single, in-depth interviews - Grounded theory analysis	15 (0:15)	32–78	PH, P, S, E
9	Covelli, V., Sattin, D., Giovannetti, A. M., Scaratti, C., Willems, M., & Leonardi, M. (2016). Caregiver's burden in disorders of consciousness: a longitudinal study. *Acta Neurol Scand, 134*(5), 352-359.	Italy	To longitudinally evaluate the multifaceted (i.e. financial, physical and psychosocial) burden of caregivers of patients with DOC.	Journal article	Quantitative study - observational (longitudinal)	4 self-reported questionnaires: SF-12, FSQ, BDI-II & COPE	216 (77:129 - 10 missing)	19–80	PH, P, S, E
10	Crawford, S., & Beaumont, J. G. (2005). Psychological needs of patients in low awareness states, their families, and health professionals. *Neuropsychol Rehabil, 15*(3-4), 548-555.	UK	To discuss the psychological reactions of relatives of patients in low awareness states.	Journal article	Commentary	Summary of research findings and staff observations	Not reported	Not reported	P, S, E

11	Crispi, F., & Crisci, C. (2000). Patients in persistent vegetative state . . . and what of their relatives? Comment. *Nursing Ethics, 7*(6), 533-535.	Italy	To highlight the burden on families associated with having a family member in a PVS.	Journal article	Commentary	Reflections on daily dialogue with relatives of PVS patients on neurorehabilitation ward	Not reported	Not reported	P, S, E
12	Crow, L. (2006). Extreme measures: a personal story of letting go. *Death Studies, 30*(2), 177-186.	USA	To provide a personal account of the author's experiences with her brother's brain injury and resulting condition.	Journal article	Commentary	Self-reported account of personal experiences	1 (0:1)	Not reported	P, S, E
13	Cruzado, J., & de la Morena, M. (2013). Coping and distress in caregivers of patients with disorders of consciousness. *Brain Injury, 27*(7-8), 793-798.	Spain	To study the depression, anxiety, and maladjustment in caregivers of patients in DOC and their relationship with coping strategies.	Journal article	Quantitative study - observational (cross-sectional)	4 self-reported questionnaires: BAI, BDI-II, Brief COPE & Maladjustment Scale (Escala de inadaptacion)	53 (12:41)	21–78	PH, P, E
14	de la Morena, M., & Cruzado, J. (2013). Caregivers of patients with disorders of consciousness: Coping and prolonged grief. *Acta Neurologica Scandinavica, 127*(6), 413-418.	Spain	To study the coping strategies and PGD of caregivers of hospitalized patients in VS or MCS and to determine the predictive value of coping for PGD.	Journal article	Quantitative study - observational (cross-sectional)	2 self-reported questionnaires: PG-12 & Brief COPE	53 (12:41)	21–78	P, E
15	Elliott, K., & McVicar, A. (2018). The impact of prolonged disorders of consciousness on the occupational life of family members. *Neuropsychological Rehabilitation, 28*(8), 1375-1391.	UK	To explore the longitudinal process of occupational adaptation within a group of primary caregivers.	Journal article	Mixed methods study - observational (cross-sectional)	24hr time diary (completed at 30 min intervals for 2 days)<br>1 self-reported questionnaire: designed for study to explore views and use of time<br>Semi-structured interviews - Burnard's analysis	6 (not reported)	18-65	PH, P, S, E
16	Giovannetti, A., Covelli, V., Sattin, D., & Leonardi, M. (2015). Caregivers of patients with disorder of consciousness: Burden, quality of life and social support. *Acta Neurologica Scandinavica, 132*(4), 259-269.	Italy	To evaluate caregivers' QOL, psychological burden and level of perceived social support, and to assess which variables may act as predictors of QOL.	Journal article	Quantitative study - observational (cross-sectional)	8 self-reported questionnaires: socio-demographic questionnaire, WHOQOL-BREF, STAI-Y, BDI-II, PG-12, COPE, STAXI-2 & MOS-SSS	129 (41:88)	Mean 52.81 (SD 13.05)	PH, P, S, E

17	Giovannetti, A. M., Leonardi, M., Pagani, M., Sattin, D., & Raggi, A. (2013). Burden of caregivers of patients in Vegetative state and minimally conscious state. *Acta Neurol Scand, 127*(1), 10-18.	Italy	To investigate perceived burden, and factor affecting perceived burden, in relatives of people diagnosed with VS and MCS.	Journal article	Quantitative study - observational (cross-sectional)	6 self-reported questionnaires: FSQ, COPE, CNA, SF-12, BDI-II & STAI-Y	487 (139:337 - 11 missing)	Mean 52.3 (SD 13.09)	PH, P, S, E
18	Giovannetti, A., Cerniauskaite, M., Leonardi, M., Sattin, D., & Covelli, V. (2015). Informal caregivers of patients with disorders of consciousness: Experience of ambiguous loss. *Brain Injury, 29*(4), 473-480.	Italy	To better understand the lived experience of caregivers and to identify key points of the psychological process of dealing with daily experience of having a relative in VS or MCS.	Journal article	Qualitative study	Semi-structured individual interviews - grounded theory analysis	20 (5:15)	32-74	PH, P, S, E
19	Giovannetti, A. M., Pagani, M., Sattin, D., Covelli, V., Raggi, A., Strazzer, S., . . . Leonardi, M. (2012). Children in vegetative state and minimally conscious state: patients' condition and caregivers' burden. *ScientificWorldJournal, 2012*, 232149. doi:10.1100/2012/232149	Italy	To evaluate caregivers' health state, coping strategies and anxiety and depression levels, and the relationship of these factors with children's level of disability.	Journal article	Quantitative study - observational (cross-sectional)	5 self-reported questionnaires: sociodemographic questionnaire, COPE, SF-12, BDI-II & STAI-Y	35 (5:30)	Mean 38.7 (SD 6.7)	PH, P, S, E
20	Gooshki, H. S., Kalkhoran, S. H. A., Ahmadi, S. M. M., Farahani, A. J., & Mahmoudi, N. (2019). The experience and reactions reported by family caregivers of vegetative patients: Qualitative Content Analysis. *International Journal of Ayurvedic Medicine, 10*(2), 162-170.	Iran	To discover the consequences experienced by caregivers of patients in a VS.	Journal article	Qualitative study	Semi-structured, face-to-face interviews - qualitative content analysis	8 (2:6)	35-58	P, S, E
21	Goudarzi, F., Abedi, H., Zarea, K., & Ahmadi, F. (2015). Multiple Victims: The Result of Caring Patients in Vegetative State. *Iran Red Crescent Med J, 17*(6), e23571.	Iran	To explore the effects of caring for patients in a VS at home on families and caregivers.	Journal article	Qualitative study	Unstructured, face-to-face interviews - content analysis	16 (5:11)	Mean 31.19	PH, P, S, E
22	Guarnerio, C., Prunas, A., Della Fontana, I., & Chiambretto, P. (2012). Prevalence and comorbidity of prolonged grief disorder in a sample of caregivers of patients in a vegetative state. *Psychiatric Quarterly, 83*(1), 65-73.	Italy	To analyse the prevalence of PGD, identify risk factors for developing PGD and investigate the association between PGD, PTSD and depression in a sample of	Journal article	Quantitative study - observational (cross-sectional)	5 self-reported questionnaires: PG-12, QD, DTS, a sociodemographic questionnaire & SCID I	40 (9:31)	31-84	P

			caregivers of VS/MSC patients hospitalized in long term care units.						
23	Hamama-Raz, Y., Zabari, Y., & Buchbinder, E. (2013). From hope to despair, and back: Being the wife of a patient in a persistent vegetative state. *Qualitative Health Research, 23*(2), 231-240.	Israel	To understand the implications and the meaning of PVS among wives providing the caregiving role.	Journal article	Qualitative study	Semi-structured, face-to-face interviews - phenomenological analysis	12 (0:12)	37-83	PH, P, S, E
24	Huber, B. (2012). What Is It Like To Live With Patients In A Vegetative State? The Psycho-Social Situation Of Parents With A Child/Youth In A Vegegative State - A Caregivers Perspective. *Brain Injury, 26*(4-5), 383-384.	Germany	To reflect on the experiences of The Lumia Foundation in providing counselling support for families who take care of their children at home.	Conference abstract	Commentary	Reflections on experiences working with family members	Not reported	Not reported	PH, P, S, E
25	Illman, N. A., & Crawford, S. (2018). Late-recovery from "permanent" vegetative state in the context of severe traumatic brain injury: A case report exploring objective and subjective aspects of recovery and rehabilitation. *Neuropsychological Rehabilitation, 28*(8), 1360-1374.	UK	To give a patient-centred and patient-focused account of a late emergence from PVS.	Journal article	Case study	1 semi-structured interview with caregiver (presented with full patient case study)	1 (0:1)	Not reported	P
26	Kitzinger, C., & Kitzinger, J. (2014). Grief, anger and despair in relatives of severely brain injured patients: Responding without pathologising. *Clinical Rehabilitation, 28*(7), 627-631.	UK	To highlight the depth and range of emotional reactions commonly experienced by families with a severely brain injured relative.	Journal article	Commentary	Reflections on interviews given by family members	51 (not reported)	Not reported	P, S, E
27	Leonardi, M., Giovannetti, A., Pagani, M., Raggi, A., & Sattin, D. (2012). Burden and needs of 487 caregivers of patients in vegetative state and in minimally conscious state: Results from a national study. *Brain Injury, 26*(10), 1201-1210.	Italy	To evaluate caregiver burden (including psychosocial difficulties, health condition, financial and socio-demographic aspects) in a large sample of caregivers of patients in a VS and MCS.	Journal article	Quantitative study - observational (cross-sectional)	7 self-reported questionnaires: CNA, FSQ, SF-12, STAI-Y, BDI-II, PG-12 & COPE	487 (139:33 - 11 missing)	18-50+	PH, P, S, E

28	Li, Y.-H., & Xu, Z.-P. (2012). Psychological crisis intervention for the family members of patients in a vegetative state. *Clinics, 67*(4), 341-345.	China	To describe the psychological stress experienced by family members of patients in a VS and evaluate the effectiveness of a psychological crisis intervention.	Journal article	Quantitative study - RCT	1 self-reported questionnaire: SCL-90-R (completed at baseline and 1-month after intervention)	107 (65:42)	41-68	PH, P, S
29	Lovstad, M., Solbraekke, K. N., Kirkevold, M., Geard, A., Kraby, A.-C., Hauger, S. L., & Schanke, A.-K. (2017). They survived but never came back-family life in the face of persistent disorders of consciousness after brain injury. *Brain Injury, 31*(6-7), 800-800.	Norway	To explore how family members of patients with DoC subjectively describe the processes they have lived through.	Conference abstract	Qualitative study	1 focus group interview	5 (not reported)	Not reported	P, S, E
30	Lovstad, M., Solbraekke, K. N., Kirkevold, M., Geard, A., Hauger, S. L., & Schanke, A.-K. (2018). "It gets better. It can"t be worse than what we have been through." Family accounts of the minimally conscious state. *Brain Injury, 32*(13-14), 1659-1669.	Norway	To explore family life and experiences of the health care system when a family member is in an MCS.	Journal article	Qualitative study	1 semi-structured focus group interview - inductive thematic analysis	5 (1:4)	20–68	P, S, E
31	Magnani, F. G., Leonardi, M., & Sattin, D. (2020). Caregivers of people with disorders of consciousness: which burden predictors? *Neurol Sci*.	Italy	To identify what factors are related to the development of mood and stress-related disorders in caregivers of patients with DoCs.	Journal article	Quantitative study - observational (cross-sectional)	5 self-reported questionnaires: sociodemographic questionnaire, BDI-II, STAI-Y, STAXI-2 & PG-12	114 (34:80)	Mean 53 (SD 13.21)	PH, P, E
32	Martone, M. (2000). Making health care decisions without a prognosis: life in a brain trauma unit. *Annu Soc Christ Ethics, 20*, 309-327.	USA	To highlight three factors where traditional ethical theory was not sufficiently nuanced to guide the author's practical decision making regarding her daughter's health care.	Journal article	Commentary	Reflection on personal experiences as a family member of a patient in a VS	1 (0:1)	Not reported	PH, P, S, E
33	Moretta, P., Estraneo, A., De Lucia, L., Cardinale, V., Loreto, V., & Trojano, L. (2014). A study of the psychological distress in family caregivers of patients with prolonged disorders of consciousness during in-hospital	Italy	To describe the social and psychological impact on primary caregivers whose relatives are affected by PDoC, and to assess evolution of such disorders	Journal article	Quantitative study - observational (longitudinal)	9 self-reported questionnaires: BDI-II, PG-12, STAI-Y, QPF/FR, COPE, CNA, MOS-SSS & FSQ (completed at baseline, 4m and 8m)	24 (9:15)	Mean 47.39 (SD 14.86)	PH, P, S, E

	rehabilitation. *Clinical Rehabilitation, 28*(7).		during the in-hospital rehabilitative phase.						
34	Moretta, P., Estraneo, A., De Lucia, L., Loreto, V., & Trojano, L. (2014). The evolution of psychological distress in caregivers of patients with prolonged disorders of consciousness during in-hospital rehabilitation. *Brain Injury, 28*(5-6), 777-777.	Italy	To investigate psychological distress over time in primary caregivers whose relatives are affected by PDoC during the in hospital rehabilitative phase.	Conference abstract	Quantitative study - observational (longitudinal)	Self-reported questionnaires: assessing depressive symptoms, state and trait anxiety, psychophysiological disturbances, prolonged grief disorder, psychological coping strategies, quality of perceived needs, perceived social support and caregiver burden (completed at baseline, 4m and 8m).	24 (9:15)	33-62	P
35	Moretta, P., Masotta, O., Crispino, E., Castronovo, G., Ruvolo, S., Montalbano, C., . . . Estraneo, A. (2017). Psychological distress is associated with altered cognitive functioning in family caregivers of patients with disorders of consciousness. *Brain Injury, 31*(8), 1088-1093.	Italy	To analyse the possible presence of reduced cognitive efficiency in association with psychological distress in the caregivers of patients in a DOC.	Journal article	Quantitative study - observational (case-control)	6 self-reported questionnaires: BDI-II, STAI-Y, QPF/FR, PG-12, FSQ & WHOQOL-brief<br>5 neuropsychological assessments: verbal fluency, Stroop brief, TMT, RAVLT, SSL-CT (compared to matched controls)	27 (9:18)	Mean 49.84 (SD 10.9)	PH, P, S, E
36	Mwaria, C. B. (1990). The concept of self in the context of crisis: A study of families of the severely brain-injured. *Social Science & Medicine, 30*(8), 889-893.	USA	To highlight the socially ambiguous and isolated positions experienced by families of patients in coma and PVS living in the greater New York area.	Journal article	Case study	Observations of participant and in-depth unstructured personal interviews	2 (1:1)	Not reported	PH, P, S, E
37	Noohi, E., Peyrovi, H., Goghary, Z. I., & Kazemi, M. (2016). Perception of social support among family caregivers of vegetative patients: A qualitative study. *Consciousness and Cognition, 41*, 150-158.	Iran	To explore the perceptions of social support among family caregivers of patients in a VS.	Journal article	Qualitative study	Face-to-face, semi-structured interviews	12 (3:9)	20-54	S, E

38	Oliveira, J. d. S., Rocha, R. M., Nery, A. A., & Constancio, J. F. (2020). Repercussions of motocyclical accident in the life of workers and their families. *Revista De Pesquisa-Cuidado E Fundamental Online, 12*, 95-101.	Brazil	To report the repercussions of a motorcycle accident on both the worker's life and his relatives.	Journal article	Case study	1 semi-structured interview with caregiver (presented with full patient case study)	1 (0:1)	Not reported	PH, P, S, E
39	Pagani, M., Giovannetti, A. M., Covelli, V., Sattin, D., & Leonardi, M. (2014). Caregiving for patients in vegetative and minimally conscious states: perceived burden as a mediator in caregivers' expression of needs and symptoms of depression and anxiety. *J Clin Psychol Med Settings, 21*(3), 214-222.	Italy	To understand the mechanisms by which DOC patients' informal caregivers' expression of their own needs is influenced by their perceived burden and symptoms of depression and anxiety.	Journal article	Quantitative study - observational (cross-sectional)	5 self-reported questionnaires: BDI-II, STAI-Y, CNA, FSQ & sociodemographic questionnaire	397 (131:266)	Mean 51.5 (SD 12.9)	P
40	Pagani, M., Giovannetti, A., Covelli, V., Sattin, D., Raggi, A., & Leonardi, M. (2014). Physical and mental health, anxiety and depressive symptoms in caregivers of patients in Vegetative State and minimally conscious state. *Clinical Psychology & Psychotherapy, 21*(5), 420-426.	Italy	To assess the relationships between personal and psychological factors of male and female caregivers of patients in VS and MCS.	Journal article	Quantitative study - observational (cross-sectional)	3 self-reported questionnaires: BDI-II, STAI-Y & SF-12	418 (124:294)	Mean 52.06	PH, P
41	Romaniello, C., Farinelli, M., Matera, N., Bertoletti, E., Pedone, V., & Northoff, G. (2015). Anxious attachment style and hopelessness as predictors of burden in caregivers of patients with disorders of consciousness: A pilot study. *Brain Injury, 29*(4), 466-472.	Italy	To investigate the relationship between attachment style, hopelessness, and overall burden of caregivers of patients in VS and MCS.	Journal article	Quantitative study - observational (cross-sectional)	3 self-reported questionnaires: CBI, ASQ & BHS	19 (4:15)	Mean 55.85 (SD 10.91)	P, S
42	Romaniello, C., Simoni, C., Farinelli, M., Bertoletti, E., Pedone, V., & Northoff, G. (2016). Emotional burden, quality of life, and coping styles in care givers of patients with disorders of consciousness living in Italy: Preliminary data. *Brain Impairment, 17*(3), 254-264.	Italy	To describe the emotional burden, quality of life, and coping styles of caregivers of patients with DOCs hospitalized in a long-term ward. To explore the relationships between these variables and their associations with socio-demographic features, and	Journal article	Quantitative study - observational (longitudinal)	3 self-reported questionnaires: FSQ, SF-36 & COPE-NVI (completed at baseline, 6m and 12m)	15 (1:14)	26–66	PH, P, S, E

			to describe their changes over time.						
43	Schembs, L., Jox, R. J., & Kuehlmeyer, K. (2018). Social Uncertainty in Disorders of Consciousness: Shedding Light on the Various Perspectives of Family Caregivers and Surrogates. *AJOB Neuroscience, 9*(2), 85-87.	Germany	To highlight the impact of social uncertainty on family members of patients in a DOC.	Journal article	Commentary	Reflections on existing research findings and theories, as well as own conversations with family members	Not reported	Not reported	P, E
44	Soeterik, S. (2017). Chapter Three: Is there a link between loss, distress, and meaning making? In S. Soeterik (PhD), *The experience of families and healthcare professionals supporting people with prolonged disorders of consciousness.* (pp. 115-133). Royal Holloway, University of London.	UK	To investigate the symptoms reported by families of a person with a PDoC, and the most appropriate loss measure to identify family members in need of additional support. To understand how successful meaning making attempts are for families after a PDoC. To establish if there is a link between the injury, ambiguous loss, meaning making difficulties and distress.	Thesis chapter	Quantitative study - observational (cross-sectional)	9 self-reported questionnaires: demographic questionnaire, WSAS, HADS, WEMWBS, BAS, CBS, PG-12, ISLES and Perspectives on Diagnosis and Prognosis of the Person with a PDoC questionnaire (completed online)	10 (1:9)	26-76+	PH, P, S
45	Soeterik, S., Connolly, S., Playford, D., Duport, S., & Riazi, A. (2017). The psychological impact of prolonged disorders of consciousness on caregivers: A systematic review of quantitative studies. *Clinical Rehabilitation, 31*(10), 1374-1385.	UK	To investigate the range of psychological constructs studied and the standardized tools used with people closely connected to a person with a DoC, and to establish the psychological impact of having a close relationship with a person with a PDoC.	Journal article	Systematic review	Systematic review of scientific databases, inclusion critera: caregivers of PDoC patients, direct study of psychological (self-reported) variables, peer-reviewed, English. 18 studies included in review.	16-487 (not reported)	Not reported	PH, P, S, E

46	Soeterik, S., Connolly, S., & Riazi, A. (2018). "Neither a wife nor a widow": An interpretative phenomenological analysis of the experiences of female family caregivers in disorders of consciousness. *Neuropsychological Rehabilitation, 28*(8), 1392-1407.	UK	To explore female family members experiences of caregiving for a person with a DoC.	Journal article	Qualitative study	Face-to-face, semi-structured interviews – Interpretative Phenomenological Analysis	9 (0:9)	26-65	P, S, E
47	Steppacher, I., & Kissler, J. (2018). A problem shared is a problem halved? Comparing burdens arising for family caregivers of patients with disorders of consciousness in institutionalized versus at home care. *BMC Psychology, (6)*.	Germany	To compare differences in subjective care burden between family caregivers of DOC patients in institutionalized care and at home.	Journal article	Quantitative study - observational (cross-sectional)	4 self-reported questionnaires: FLZ, SVF, TF & LEBE (all completed remotely, 18 paper and pencil, 19 online)	81 (25:56)	Mean 51.33	PH, P, S, E
48	Tran, J., Scott, S., & Martinez, K. M. (2015). Veterans with Severe TBI Complicated by Disorder of Consciousness: Caregiver Stress and Needs Assessment...American Congress of Rehabilitation Medicine Annual Conference 25-30 October, 2015, Dallas, TX, USA. *Archives of Physical Medicine & Rehabilitation, 96*(12), e13-e13.	USA	To identify the needs of caregivers of veterans with severe traumatic brain injury complicated by chronic DOCs.	Conference abstract	Qualitative study	1 hour-long focus group	5 (not reported)	Not reported	P, S, E
49	Zucchella, C., Di Santis, M., Ciccone, B., Pelella, M., Scappaticci, M., Badalassi, G., . . . Bartolo, M. (2018). Is telemonitoring useful for supporting persons with consciousness disorders and caregivers? A preliminary observational study in a real-life population. *Journal of Telemedicine and Telecare, 24*(1), 56-62.	Italy	To test the feasibility of home-care assistance telemonitoring system for supporting patients in a DOC at home and investigate the impact of the system on caregivers' quality of life and psychological wellness.	Journal article	Quantitative study - interventional (longitudinal)	3 self-reported questionnaires: WHOQOL-BREF, HDRS & HARS (completed by caregiver at baseline and 12m - global satisfaction 12m only, presented alongside patient intervention assessments)	Not reported	Not reported	PH, P, S, E
50	Shilansky, A., & Weitz, R. (2002). Treating families of patients in vegetative state: Adjustment and interaction with hospital staff. In G. Dolce & L. Sazbon (Eds.), *The Post-traumatic Vegetative State*. Thieme: Stuttgart.	Israel	To present a broader perspective on the complex and intertwined undercurrents that make up the world of unconscious patients and their families, in their daily interactions and encounters with staff members.	Book chapter	Commentary	Overview (not systematic) of current research literature	Not reported	Not reported	PH, P, S, E

Abbreviation key: ADSS (Anxiety and Depression Short Scale), ASQ (Attachment Style Questionnaire), BAI (Beck Anxiety Inventory), BAS (Boundary Ambiguity Scale), BDI-II (Beck Depression Inventory), BHS (Beck Hopelessness Scale), CBI (Caregiver Burden Inventory), CBS (Continuing Bonds Scale), CISS (Coping Inventory for Stressful Situations), CNA (Caregiver Needs Assessment), COPE (Coping Orientations to Problem Experiences), COPE-NVI (Coping Orientations to Problem Experiences - Nuova Versione Italiana), CQOL (Caregiver Quality of Life), DTS (Davidson Trauma Scale), E (Environmental), EPQ (Eysenck Personality Questionnaire), FLZ (Fragebogen zur Lebenszufriedenheit – Life Satisfaction Questionanire), FSQ (Family Strain Questionnaire), FSQ-SF (Family Strain Questionnaire – Short Form), HARS (Hamilton Anxiety Rating Scale), HADS (Hospital Anxiety and Depression Scale), HDRS (Hamilton Depression Rating Scale), ISLES (Inventory of Stressful Life Events Scale), LEBE (Fragebogen zu Lebensbedeutung und Lebenssinn – Purpose and Meaning of Life Questionnaire), MOS-SSS (Medical Outcome Study Social Support Survey), P (Psychological), PDoC (Prolonged Disorder of Consciousness), PG-12 (Prolonged Grief 12), PH (Physical Health), QD (Depression Questionnaire), QPF/FR (Questionario PsicoFisiologico/Forma Ridotta – Psychophysiological Inventory/Reduced Form), RAVLT (Rey Auditory Verbal Learning Test), S (Social), SCID I (Structured Clinical Interview for DSM-IV Axis I disorders), SCL-90-R (Symptom Check List-90-R), SF-12 (Short Form 12), SF-36 (Short Form 36 Health Survey), SSL-CT (Supra-span learning - Corsi's test), STAI X1 X2 (State Trait Anxiety Inventory), STAI-Y (Spielberger State-Trait Anxiety Inventory-Y), STAXI-2 (State-Trait Anger Expression Inventory-2), SVF (Stressverarbeitungsfragebogen – Coping Strategy Questionnaire), TF (Trauerfragebogen – Greif Questionnaire), TMT (Trail Making Test), WEMWBS (Warwick Edinburgh Mental Wellbeing Scale), WHOQOL-BREF (World Health Organization Quality of Life-Bref), WSAS (Work and Social Adjustment Scale.
